# Axon Diversity of Lamina I Local-Circuit Neurons in the Lumbar Spinal Cord

**DOI:** 10.1002/cne.23311

**Published:** 2013-02-05

**Authors:** Peter Szucs, Liliana L Luz, Raquel Pinho, Paulo Aguiar, Zsófia Antal, Sheena YX Tiong, Andrew J Todd, Boris V Safronov

**Affiliations:** 1 Spinal Neuronal Networks Group, Institute of Molecular and Cell Biology (IBMC), University of Porto4150-180 Porto, Portugal; 2 Faculty of Sciences, Department of Applied Mathematics, Center of Mathematics of the University of Porto (CMUP)4150-180 Porto, Portugal; 3 Department of Anatomy, Histology and Embryology, University of Debrecen, Medical and Health Science Center4032 Debrecen, Hungary; 4 Spinal Cord Group, College of Medical Veterinary and Life Sciences, University of GlasgowG12 8QQ Glasgow, United Kingdom

**Keywords:** interneuron, propriospinal connection, varicosity distribution, propagation time, 3-D reconstruction

## Abstract

Spinal lamina I is a key area for relaying and integrating information from nociceptive primary afferents with various other sources of inputs. Although lamina I projection neurons have been intensively studied, much less attention has been given to local-circuit neurons (LCNs), which form the majority of the lamina I neuronal population. In this work the infrared light-emitting diode oblique illumination technique was used to visualize and label LCNs, allowing reconstruction and analysis of their dendritic and extensive axonal trees. We show that the majority of lamina I neurons with locally branching axons fall into the multipolar (with ventrally protruding dendrites) and flattened (dendrites limited to lamina I) somatodendritic categories. Analysis of their axons revealed that the initial myelinated part gives rise to several unmyelinated small-diameter branches that have a high number of densely packed, large varicosities and an extensive rostrocaudal (two or three segments), mediolateral, and dorsoventral (reaching laminae III–IV) distribution. The extent of the axon and the occasional presence of long, solitary branches suggest that LCNs may also form short and long propriospinal connections. We also found that the distribution of axon varicosities and terminal field locations show substantial heterogeneity and that a substantial portion of LCNs is inhibitory. Our observations indicate that LCNs of lamina I form intersegmental as well as interlaminar connections and may govern large numbers of neurons, providing anatomical substrate for rostrocaudal “processing units” in the dorsal horn. J. Comp. Neurol. 521:2719–2741, 2013.

Lamina I of the spinal cord is a key area for sensory information processing and pain transmission (Cervero and Tattersall, 1987; Christensen and Perl, 1970). It is a major target zone for the fine-caliber myelinated Adelta- and unmyelinated C-primary afferent fibers (Willis and Coggeshall, 1991) as well as for the descending systems that control its activity (Millan, 2002). Based on their somatodendritic organization, lamina I neurons in the rat have been classified as fusiform (IA and IB), multipolar (IIA and IIB), flattened (III), or pyramidal (IV; Lima and Coimbra, 1986). However, as for the rest of the dorsal horn, and in particular lamina I neurons, little is known about the local axonal projections, and the lack of such information is a serious obstacle to establishing the roles of different neurons and understanding the dorsal horn circuitry (Todd, 2010). It is important to note that only 5% of lamina I neurons project supraspinally, whereas the majority of lamina I neurons function as excitatory and inhibitory intrinsic, or so-called local-circuit, spinal neurons (Bice and Beal, 1997a,b; Cervero et al., 1979; Dickenson et al., 1997; Grudt and Perl, 2002; Hunt et al., 1981; Spike et al., 2003). Hence, lamina I neurons were shown to issue collaterals in laminae I–IV of the spinal cord in monkey (Beal et al., 1981), cat (Bennett et al., 1981; Hylden et al., 1986; Light et al., 1979), and rat (Cheunsuang and Morris, 2000; Grudt and Perl, 2002) as well as in the medullary dorsal horn of rats (Li et al., 2000). However, a systematic study on the functional connectivity of these neurons or the branching pattern and extent of their axons has not been performed yet. This most likely is due to the low yield of available techniques for labeling and reconstructing intact single neurons in lamina I.

The use of the infrared light-emitting diode (IR-LED) oblique illumination technique (Safronov et al., 2007; Szucs et al., 2009) in intact spinal cord preparations, in vitro, has proved to be a way to solve this problem. This approach permits recording, labeling, and reconstruction of the complete dendritic and axonal trees of lamina I neurons, revealing distinct local axon-collateral patterns for projection neurons belonging to the anterolateral tract (ALT; Szucs et al., 2010). These experiments also revealed lamina I neurons with extensive local axons (see Fig. 3. of Szucs et al., 2010) similar to those reported by Li et al. (2000) in the medullary dorsal horn. In a recent study, we used a computer model of a 3-D reconstructed LCN to show that such complex axon architecture may significantly contribute to long transmission delays in local monosynaptic connections (Luz et al., 2010). Thus, our aim in the present study was to provide a detailed morphological description of lamina I LCNs, with special emphasis on the axon structure, in order to improve our understanding of their role in the spinal dorsal horn network. We also sought to create the first detailed 3-D reconstructions of lamina I neurons that, in addition to being a valuable tool for computational neuroscience, would also allow novel, spatially dependent morphometric measurements.

## MATERIALS AND METHODS

### Spinal cord preparation

Laboratory Wistar rats (P14–P24) were killed in accordance with the national guidelines (Direcção Geral de Veterinária, Ministério da Agricultura) after anesthesia with an intraperitoneal injection of Na^+^-pentobarbital (30 mg/kg) and subsequent check for lack of pedal withdrawal reflexes. The vertebral column was quickly cut out and immersed in oxygenated artificial cerebrospinal fluid (ACSF) at room temperature. The lumbar spinal cord was dissected, and the pia mater was locally removed in the region of interest with forceps and scissors to provide access for the recording pipettes. The spinal cord was glued with cyanoacrylate adhesive to a golden plate with the dorsolateral spinal cord surface facing upward and transferred to the recording chamber. All recordings were performed at 22–24°C.

### Segment length measurements

Lumbar spinal cords of P14 (n = 3) and P21 (n = 3) rats were excised as described above and immersion fixed in 4% paraformaldehyde to achieve a fixation comparable to that of the spinal cords used in electrophysiological experiments and for cell labeling. The rostrocaudal extent of the spread of rootlets from a given dorsal root was measured with a Vernier caliper for all segments.

### Imaging and identification of lamina I neurons

**Figure 1:**
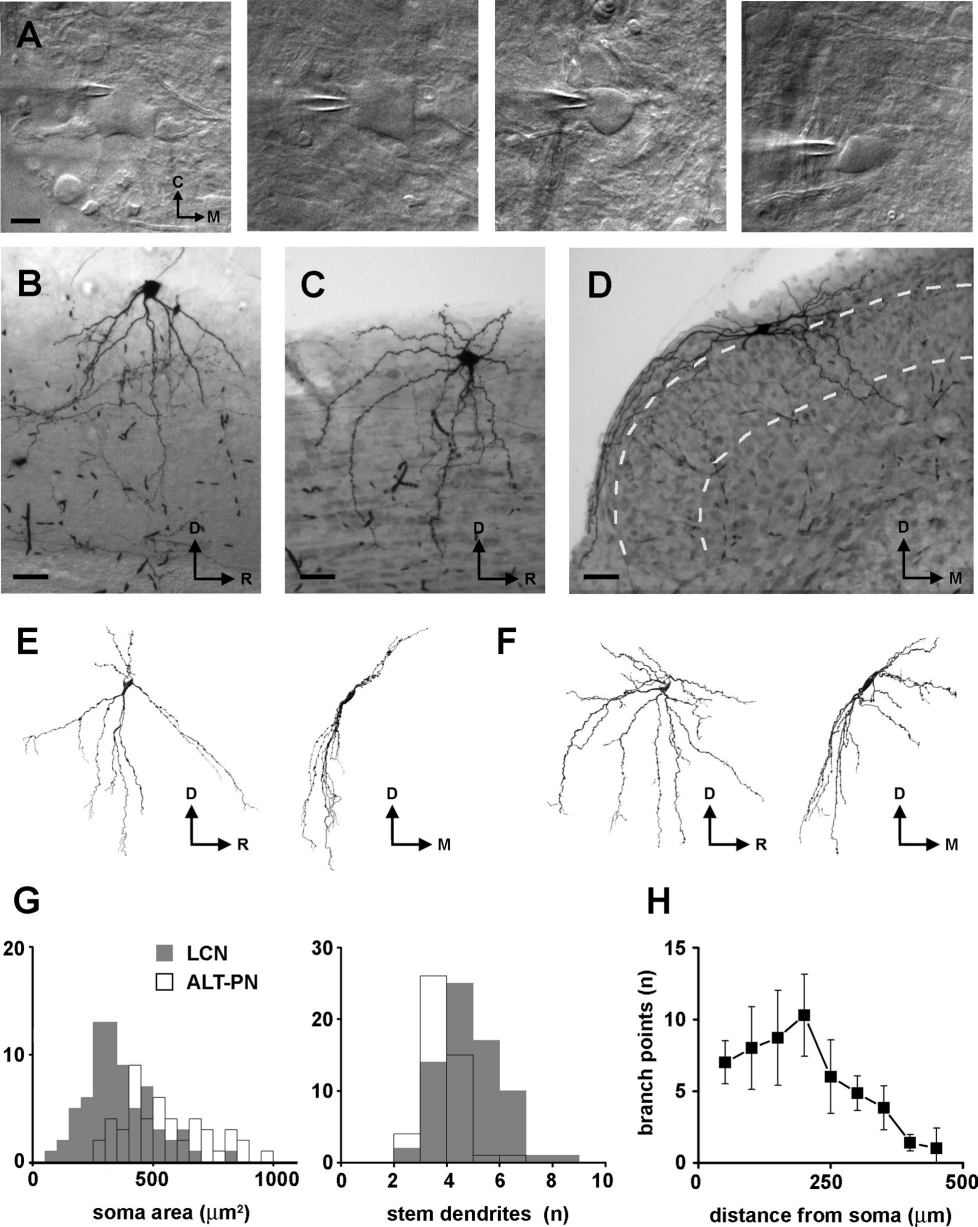
Somatodendritic features of LCNs. **A**: Images of large (left pair) and smaller (right pair) LCNs during the process of cell labeling. **B**: Photomicrograph of the soma, dendrites, and axon branches of a typical flattened LCN in a sagittal spinal cord section. **C**: Photomicrograph of a typical multipolar LCN in a sagittal section. **D**: Another multipolar LCN in a transverse section. Dashed line indicates the rough borders of lamina II. The section is slightly distorted because of detachment from the supporting agar during processing. **E**: Sagittal (left) and transverse (right) rotated views of a 3-D reconstructed flattened LCN (reconstruction from sagittal sections; axon omitted for clarity). **F**: Sagittal (left) and transverse (right) rotated views of a 3-D reconstructed multipolar LCN (reconstruction from sagittal sections; axon omitted for clarity). **G**: Histograms of soma area distribution (left) and number of stem dendrites (right) in LCNs (shaded bars) and ALT-PNs (open bars). The data for the ALT-PNs are from Szucs et al. (2010). **H**: 3-D Sholl analysis of the number of dendritic branch points in 50-μm-thick shells. C, caudal; M, medial; D, dorsal; R, rostral (throughout the figures). Bin sizes in G = 50 μm^2^ in the soma area histogram; 1 in the stem dendrite histogram. Scale bars = 20 μm in A; 50 μm in B–D.

Lamina I neurons were visualized in the intact lumbar spinal cord using the oblique IR-LED illumination technique (Safronov et al., 2007; Szucs et al., 2009). Neurons were selected in the region between the dorsolateral funiculus (lateral border) and the dorsal root entry zone (medial border; see Fig. 1. of Pinto et al., 2010). The white matter covering this part of lamina I is thin in young rats, allowing visually controlled, tight-seal recordings from the superficial neurons. Neurons with multipolar or rounded somata were selected (Fig. 1A) because these somatic morphologies were unlike those of ALT-PNs described in our previous studies (Szucs et al., 2010; Luz et al., 2010). Only cells in the most superficial cell layer, with an apparent soma diameter above 20 μm, were selected for recording.

### Cell recording and filling

Recordings from lamina I neurons were made in whole-cell mode. The ACSF contained (in mM): NaCl 115, KCl 3, CaCl_2_ 2, MgCl_2_ 1, NaH_2_PO_4_ 1, NaHCO_3_ 25, and glucose 11 (pH 7.4 when bubbled with 95%–5% mixture of O_2_–CO_2_). Pipettes were pulled from thick-walled glass (BioMedical Instruments) and fire polished (resistance 3–5 MΩ). The pipette solution contained (in mM): KCl 3, K-gluconate 150, MgCl_2_ 1, BAPTA 1, HEPES 10 (pH 7.3 adjusted with KOH, final [K^+^] was 160 mM), and 0.5–1% biocytin. In the case of neurons that underwent further immunocytochemical analysis, the 0.5% biocytin was complemented by 0.5% rhodamine red. Recordings were made with an EPC10-double amplifier (HEKA, Lambrecht, Germany). The signal was lowpass filtered at 2.9 kHz and sampled at 10 kHz. Offset potentials were compensated before seal formation. Liquid junction potentials were calculated (15.9 mV) and corrected for in all experiments using the compensation circuitry of the amplifier. In some experiments, substance P (SP; Sigma, St. Louis, MO) was applied in the bath through the perfusion line. Application of SP was never repeated in the same preparation.

### Visualization of filled neurons and measurements

After fixation in 4% freshly depolymerized formaldehyde, the spinal cord was embedded in agar, and 100-μm-thick sagittal or transverse serial sections were prepared with a tissue slicer (Leica VT 1000S). To reveal biocytin, sections were permeabilized with 50% ethanol and treated according to the avidin-biotinylated horseradish peroxidase (HRP) method (ExtrAvidin-Peroxidase, diluted 1:1,000; Sigma), followed by a diaminobenzidine (DAB) chromogen reaction. Sections were either counterstained on slides with 1% toluidine blue, dehydrated, cleared, and coverslipped with DPX (Fluka, Buchs, Switzerland) or treated with 1% OsO_4_ and embedded in epoxy resin (Durcupan; Fluka). Photomicrographs were taken using the ×10 or ×40 dry lens of a Primo Star (Zeiss) microscope equipped with a Guppy (Allied Vision Technologies) digital camera. In some cases, multiple (three to six) photographs were taken from consecutive focal planes and overlaid in order to see focused details from different depths. These photographs are referred to as “extended focal images” in the illustrations. Contrast and brightness of the photographic images used in all the figures were adjusted in Adobe Image Ready software.

The areas of filled neuronal somata were measured using the ImageTool software (University of Texas Health Science Center at San Antonio) by tracing the outline of the cell body with a mouse on digital images taken with a ×10 objective lens. In the case of 3-D reconstructed and connected cells, the largest extent of the axonal and dendritic tree in each direction was measured on the reconstructions. For cells that were not reconstructed and connected, the measurements were performed on digital photographs or by overlaying a dimmed scale bar on the live image of the camera connected to the microscope. In sagittal sections, the rostrocaudal extent could be directly measured, and the mediolateral extent could be estimated by multiplying the number of sections that had labeled processes by the thickness of the sections. The dorsoventral extent in sagittal sections, especially in lateral ones, could not be measured reliably because of the curvature of the dorsal horn. In transverse sections, the mediolateral and dorsoventral extent could be directly measured, whereas the rostrocaudal extent was estimated in the same way as the mediolateral extent for sagittal sections.

Because of the difficulty of exact delineation of laminae in lateral sagittal sections, laminar borders are not indicated on reconstructions from sagittal sections even when they are rotated to show a transverse view (e.g., Fig. 1E,F). Laminar location of axon branches was estimated by checking their distance from the dorsal surface (in medial sagittal sections, in which the dorsal horn curvature introduces less error) and from other landmarks, such as the notch at the level of the neck of the dorsal horn or the central canal. When these landmarks were located in more medial serial sections, photographic images of the sections were overlaid for alignment.

### 3-D reconstruction

Complete 3-D reconstructions were performed from serial sections with Neurolucida (MBF Bioscience, Williston, VT). We found that the greatest extent of the axonal tree of LCNs is in the rostrocaudal direction, so we chose cells sectioned in the sagittal plane for reconstruction. Because of this, there were fewer connections to be made between neighboring sections.

First, each section was completely traced onto the corresponding section of a serial section data set with a ×40 (dry) objective. Caliber of the digitally traced processes was continuously adjusted during the tracing to cover completely the video image of the labeled process. Fiber caliber units for the selected lens were automatically set by the software, based on prior calibration. Whenever necessary, a ×100 (oil immersion) lens was used to determine the Z difference between crossing structures. Neighboring sections were not aligned, nor were continuing processes connected at this point. Next, we aligned the sections and connected the pieces working always toward the section containing the soma. As a result of shrinkage, the thickness (Z dimension) of resin-embedded sections was 80–90% of the original 100 μm. This was comparable to shrinkage of these long sagittal spinal cord sections along the X–Y axes. Thus, Z shrinkage was not corrected in these cases. In the case of DPX-embedded material, the shrinkage along the Z axis was corrected to reach a thickness of 80 μm and to be comparable to the other reconstructions. In most cases, tracing and the procedure of alignment followed by connecting the corresponding pieces were performed by independent persons, to ensure that all difficult branch points and crossings were double checked. Section contours as well as gray matter and central canal borders were traced at the bottom level of each section. Neuronal processes that could not be connected because of partial filling, distortion of the sections, or any other technical problems were deleted from the data set. The estimated percentage of these deleted processes was below 5% in all cases.

### Calculation and 3-D visualization of varicosity distributions and action potential propagation time maps

A set of specific functions (called Py3DN; https://sourceforge.net/projects/py3dn/) was developed in the Python programming language to perform particular, spatially dependent morphometric analyses on the axonal trees. The Neurolucida data of the reconstructed axon were made available to the Python environment using a custom-made parser. The morphometric analysis algorithms were integrated into Blender, a well-established, free, open source 3-D content creation suite (http://www.blender.org), to visualize the spatially dependent results.

For the visual representation of varicosity distribution along the axonal tree of LCNs, a virtual boundary box that encompasses the entire axonal tree was calculated. This volume was partitioned into voxels (or volume elements) with a predefined size (100 μm × 100 μm × 100 μm). The number of varicosities in each voxel was then calculated, and its normalized value was used to color-code the particular voxel. For better visualization of the overlapping voxels, the opacity index of every voxel was also set to the normalized value of its varicosity content.

For creating propagation time maps along the LCN axonal trees, the time needed for a hypothetical action potential, initiated at the initial segment, to reach each point of the axon was calculated. The propagation time contribution of each axon segment (i.e., between two points of the reconstruction) was assumed to depend on its length and diameter. A diameter threshold based on electron microscopic measurements (see Fig. 7) was used to distinguish between myelinated and unmyelinated elements of the axon. The equation used for calculating the propagation time δ_m_ of myelinated regions was δ_m_ = l/(k_m_d), where l is length, k_m_ is a constant factor, and d is the diameter. The constant k_m_ was 10,000 m/second · μm^−1^, giving 10 m/second conduction velocity for a uniform 1-μm-thick constant-diameter axon (Cervero et al., 1979). On the other hand, the equation used for calculating the propagation time δ_n_ for unmyelinated elements was δ_n_ = l/(k_n_d), where l is the element length, k_n_ is a constant factor, and d is the diameter. The constant k_n_ was chosen to be 380 m/second · μm^−1/2^, which implies a conduction velocity of 0.38 m/second in a 1-μm-thick unmyelinated axon. This value was in good agreement with conduction velocities measured at 22–24°C for unmyelinated C-fibers in isolated dorsal roots (Pinto et al., 2008). The cumulative propagation time for each axon point was calculated as the sum of propagation times of all elements up to the axon initial segment. A color code was used to represent the cumulative propagation time at each point of the axon. Because of the lack of data on the length of the axon initial segment of lamina I neurons, this part was treated as the rest of the axon tree. Sholl analysis, path distance calculation, and basic quantitative measurements were performed by Neuroexplorer (MBF Bioscience).

### Immunocytochemistry

Spinal cords containing neurons filled with a mixture of biocytin and rhodamine for immunocytochemistry were fixed in 4% formaldehyde for at least 24 hours at 4°C. Serial sections of 60–100 μm thickness were prepared, in the sagittal or transverse plane, and a Zeiss AxioLab.A1 fluorescence microscope was used to identify the section containing the cell body. This section and its two immediate neighbors were treated with a cocktail of primary antibodies consisting of rabbit antivesicular GABA transporter (VGAT; 1:1,000; Synaptic Systems, Goettingen, Germany) and guinea pig antivesicular glutamate transporter 2 (VGLUT2; 1:5,000; Millipore, Watford, United Kingdom), as described previously (Yasaka et al., 2010). Filled cells were labeled with streptavidin-conjugated fluorochrome (avidin-rhodamine; 1:1,000; Jackson Immunoresearch, West Grove, PA), and species-specific secondary antibodies raised in donkey or goat and labeled with fluorescent dyes (Alexa 488, Pacific blue, 1:500, Invitrogen, Paisley, United Kingdom; or DyLight 649, 1:100, Jackson Immunoresearch) were used to perform triple-fluorescence staining. Details of the antibodies are given in Table 1. The VGLUT2 antibody recognizes a band of 56 kDa (corresponding to the VGLUT2 protein) on Western blots of rat brain lysate. Its specificity was confirmed by carrying out dual-immunofluorescence staining with a well-characterized rabbit antibody against VGLUT2 (Todd et al., 2003), and identical structures were stained by the two antibodies (Yasaka et al., 2010). The VGAT antibody stains a band of the appropriate molecular weight on Western blots (Guo et al., 2009). Immunofluorescence staining was blocked by preincubation with the immunizing peptide at 10^−6^ M (Polgar et al., 2011).

**TABLE 1:** Details of the Antibodies Used^1^

Antibody	Host	Antigen	Amino acid sequence	Supplier	Catalog No.	Dilution
VGLUT2	Guinea pig	18 amino acids at C-terminal end of rat VGLUT2	VQESAQDAYSYKDRDDYS	Millipore	AB2251	1:5,000
VGAT	Rabbit polyclonal	amino acids 75–87 of rat VGAT coupled to KLH	AEPPVEGDIHYQR	Synaptic Systems	131 002	1:1,000

1 VGLUT2, vesicular glutamate transporter 2; VGAT, vesicular GABA transporter; KLH, keyhole limpet hemocyanin.

Sections were mounted and coverslipped with antifade medium, and series of optical sections were acquired sequentially at 0.5-μm intervals with a Zeiss LSM 710 confocal microscope through a ×63 oil-immersion lens (NA 1.4). Finally, sections were demounted and processed further with the HRP-DAB method for morphometric measurements or 3-D reconstruction (see above).

### Electron microscopy

Spinal cords processed for electron microscopic analysis were fixed in a mixture of 4% formaldehyde and 0.1% glutaraldehyde. Serial sagittal 100-μm-thick sections were cut with a tissue slicer (Leica, VT 1000S). Sections were permeabilized by freeze–thaw cycles in liquid nitrogen and the biocytin revealed by the HRP-DAB reaction, as described above. Sections were postfixed in 1% OsO_4_, dehydrated, and embedded into epoxy resin (Durcupan; Fluka) on glass slides. Regions of interest were excised from the thin layer of resin on the slide and re-embedded for ultrathin sectioning. The ultrathin sections were further contrasted with lead citrate (1 minute) and uranyl acetate (1 minute) and scanned with an electron microscope (JEM 1400 TEM; JEOL, Tokyo, Japan). Biocytin-filled axonal profiles were digitally recorded using a Gatan SC 1000 Orius CCD camera (Gatan, Warrendale, PA) at ×20,000 or ×50,000 magnification. Diameter of axons and axon varicosities was measured on the digital images. Numbers are given as mean ± SEM unless otherwise mentioned.

## RESULTS

The following experiments were performed to characterize local-circuit neurons in the most superficial layer of the spinal dorsal horn. We have recorded from and filled 98 lamina I neurons, in the intact in vitro spinal cord preparation containing at least two but in most cases all six (L1–L6) lumbar segments. In 82 cases, the axon of the recorded neuron had been recovered to an extent that allowed us to confirm 1) the lack of any axon in the contralateral white or gray matter and 2) the presence of numerous ipsilateral, local axon collaterals with varicosities. These 82 neurons form the basis of this study.

The mean input resistance of LCNs was 0.9 ± 0.1 GΩ. With the exception of a few neurons, LCNs presented a tonic firing pattern, and, in 46% of the cases, neurons were rhythmically firing action potentials (at zero injected current) that persisted in the presence of a blocker of fast glutamatergic transmission, CNQX (10 μM; Sigma; see Li and Baccei, 2011). About one-third of the LCNs tested (n = 34) responded with a characteristic inward current for the bath application of SP (1 μM; Sigma), an agonist of functional neurokinin-1 receptors. When recorded in current-clamp mode, the SP-activated current caused depolarization that was often suprathreshold and resulted in sustained action potential firing. Analysis of the electrophysiological and pharmacological properties of LCNs will be the subject of another study.

From the 82 LCNs, 60 have been sectioned in the sagittal, 22 in the transverse plane, and a total of 13 cells have been reconstructed. Complete 3-D reconstruction and connection of the reconstructed pieces was performed for seven LCNs (cell IDs: L292_E1, L292_E5, L387_E3-2, L396_E2, L420_E2, Zs022_E8-1, L571_E20-3). For three more LCNs, 3-D reconstruction was performed (cell ID: Zs079_E13 sagittal plane; cell ID: L315_E4 and L316_E6 transverse plane), but connection of the pieces could not be performed because of distortion or damage to some of the sections. Three LCNs have been fully reconstructed and aligned in 2-D (cell IDs: L256_E1 and L279_E2 sagittal plane; cell ID: L317_E1 transverse plane), similarly to the reconstructions of Szucs et al. (2010). In addition, for comparison of basic parameters of the axon, a mixed-collateral-type (MCT) ALT-PN (cell ID: L292_E4) has also been reconstructed in 3-D.

### Somatodendritic architecture of lamina I LCNs

**Figure 2:**
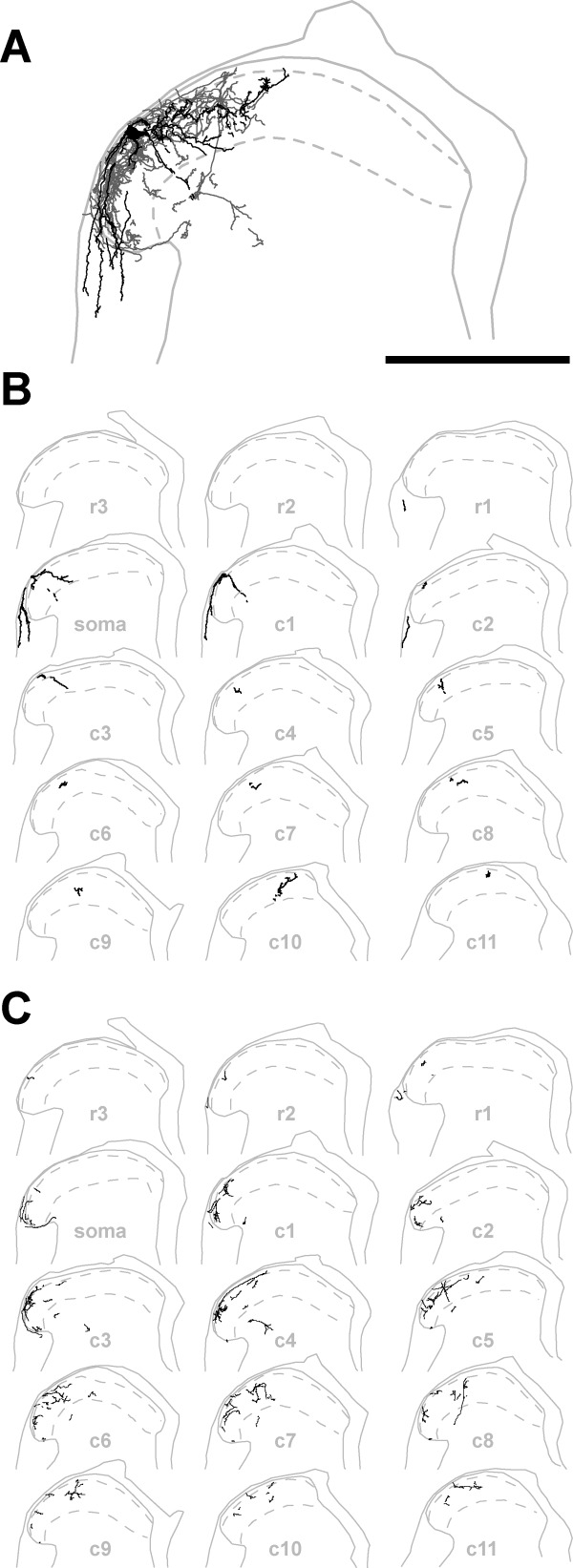
Typical multipolar LCN reconstructed from transverse serial sections. **A**: Overlaid image of 15 transverse, 100-μm-thick, serial sections. Dendrites (black) and axon (dark gray) of the LCN (cell ID: L316_E6) occupy laminae I–II and protrude into lamina III. An axon collateral descends ventrally beyond the neck of the dorsal horn, and some dendrites are located in the DLF. For clarity, borders of the white and gray matter (continuous light gray line; same in B,C) as well as of lamina II (dashed light gray line; same in B,C) are shown only for the section containing the cell body. **B**: Reconstructions showing the location of dendritic pieces (black) in the individual transverse serial sections. **C**: Reconstructions indicating the location of axonal pieces (black) in the individual transverse serial sections. soma, Section with the cell body; r, rostral; c, caudal. Scale bar = 500 μm.

Morphometric analysis of the somatodendritic domain was possible in 72 cases, in which both soma and dendrites were revealed in detail. The majority of these recovered neurons fell into the multipolar (n = 38) and flattened (n = 29) somatodendritic types of Lima and Coimbra (1986). We also found one fusiform and one pyramidal-like soma in our sample, and three neurons could not be classified according to this scheme. In the case of multipolar neurons (Fig. 1C,D,F), initial dendrites often originated close to each other, resulting in a gradual tapering appearance of the soma toward the first dendritic branch point. Some of the multipolar dendrites protruded ventrally and reached lamina III (Figs. 1D,F, 2A,B), whereas dendrites of flattened neurons were mostly restricted to lamina I and outer lamina II (Fig. 1B,E). Dendrites of LCNs had few spines compared with those of ALT-PNs in our earlier study (see Figs. 1Db and 5A of Szucs et al., 2010).

LCNs had a mean soma diameter of 22.4 ± 0.6 μm (n = 72) and gave rise to two to eight major dendrites (average 4.4 ± 0.1). In comparing LCN soma areas and number of their initial dendrites with those of ALT-PNs from our previous study (n = 40; Szucs et al., 2010), we found that the majority of LCNs were smaller (average 351 ± 17 μm^2^) and gave rise to more initial dendrites than ALT-PNs (Fig. 1G). Basic morphometric parameters of the somatodendritic domain of LCNs are shown in Table 2. The rostrocaudal extent of the dendrites of LCNs was 320 ± 14 μm, whereas mediolateral and dorsoventral spread were 323 ± 15 μm and 159 ± 19 μm, respectively. Morphometric parameters of flattened and multipolar LCNs were similar. However, multipolar LCNs often gave rise to more dendrites, and the dorsoventral extent of their dendrites, in line with the original description of lamina I multipolar neurons (Lima and Coimbra, 1986), was greater than that of flattened cells. The single fusiform and the pyramidal LCN along with the cells that could not be classified had larger dendritic trees. Sholl analysis of the branch points (n = 7; reconstructed in 3-D) revealed that dendrites of LCNs branched mostly in the vicinity (50–250 μm) of the soma (Fig. 1H). We found no indication for any correlation between the somatodendritic type and investigated axon parameters of LCNs.

**TABLE 2:** Basic Morphometric Parameters of the Somatodendritic and Axonal Domains of LCNs1

		Soma		Dendritic tree dimensions (μm)			Axonal tree dimensions (μm)				Plane	
					
SD type	n	Area (μm^2^)	Stem	RC	ML	DV	RC	ML	DV	Cut	S	T
Flattened	29	357 ± 30 (93–679)	4.2 ± 0.2 (3–6)	301 ± 21 (150–500)	304 ± 19 (200–620)	65 ± 25 (25–110)	2,429 ± 205 (800–5,200)	656 ± 43 (400–1,100)	288 ± 40 (124–479)	18	23	6
Multipolar	38	357 ± 23 (110–839)	4.8 ± 0.2 (2–8)	323 ± 17 (140–500)	327 ± 21 (197–634)	178 ± 16 (107–250)	2,401 ± 173 (1,100–6,500)	691 ± 41 (312–1,300)	331 ± 84 (133–1,050)	22	30	8
Other + n.c.	5	261 ± 30 (172–304)	3.0 ± 0.4 (2–4)	437 ± 134 (260–700)	405 ± 105 (300–720)	260	1,868 ± 145 (700–2,700)	459 ± 38 (240–800)	248 ± 67 (120–680)	11	7	8
No soma recovered	10	n.a.	n.a.	n.a.	n.a.	n.a.						
All LCNs^2^	82	351 ± 17 (93–839)	4.4 ± 0.1 (2–8)	320 ± 14 (140–700)	323 ± 15 (197–720)	159 ± 19 (25–260)	2,312 ± 113 (700–6,500)	636 ± 27 (240–1,300)	295 ± 41 (120–1,050)	51	60	22

1 Data are presented as mean ± SEM (range). SD type, somatodendritic type determined using criteria of Lima and Coimbra (1986); n.c., not classified; n.a., not applicable for neurons when the soma was not recovered; stem, number of stem dendrites from the soma; RC, rostrocaudal; ML, mediolateral; DV, dorsoventral; cut, the axon of the cell reached one or both ends of the spinal cord preparation and, consequently, was severed; plane, sectioning plane; S, sagittal; T, transverse.

2 Neurons for which the soma was not recovered (n = 10) were excluded from the analysis of the dendritic tree.

### Axon of lamina I LCNs

**Figure 3:**
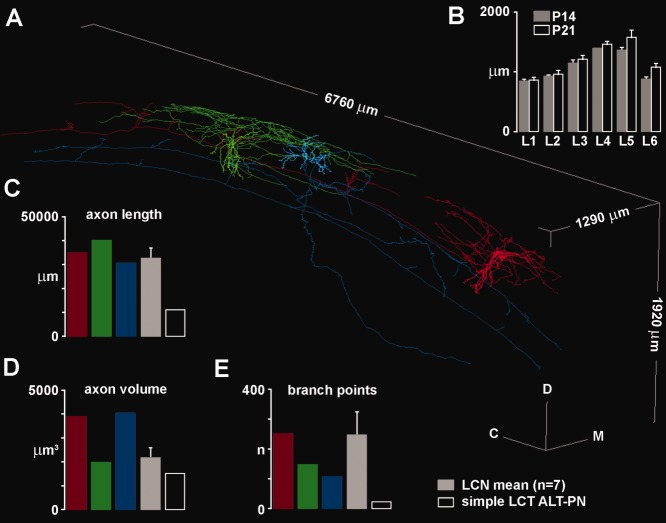
Comparison of basic axon parameters of 3-D reconstructed LCNs and ALT-PNs. **A**: 3-D reconstruction of two LCNs (cell ID: L292_E1 in red; cell ID: L292_E5 in green) and a mixed-collateral-type (MCT) ALT-PN (cell ID: L292_E4 in blue), filled in the same spinal cord. Rostrocaudal, mediolateral, and dorsoventral dimensions of the bounding box (light gray) are indicated along the corresponding axis. **B**: Length of different lumbar spinal cord segments (L1–L6) at postnatal days 14 (P14) and 21 (P21). Columns in each case show the average and standard mean error of measurements in three different animals. **C–E**: Total axon length (i.e., sum of the lengths of all reconstructed axon segments), total axon volume (i.e., sum of all reconstructed axon segment volumes, calculated from segment length and local diameter), and number of branch points along the axon of individual cells (red, green, blue, and open columns) along with the mean value for 3-D reconstructed LCNs (gray column; n = 7).

The axon of LCNs in all cases formed a denser or sparser local network that spanned at least one or two segments rostrocaudally. Axon branches occupied the dorsal 100–120-μm-thick band of the gray matter (corresponding roughly to laminae I–II at this age) and frequently protruded deeper, occasionally reaching the level of the notch at the neck of the dorsal horn (corresponding to laminae III–IV in most lumbar segments at this age and also in adult rats; Paxinos and Watson, 2007). Distribution of axon branches was also confirmed in transverse sections (Fig. 2A,C). In two cases, a few axon collaterals reached below the level of the central canal (laminae VII and VIII). The local axon network was either centered on the soma (e.g., green neuron, cell ID: L292_E5, in Fig. 3A) or shifted along the rostrocaudal axis (e.g., red neuron, cell ID: L292_E1, in Fig. 3A or cell ID: L387_E3-2, in Fig. 6D). In most cases, the axon overlapped with the dendrites and the soma. The mean rostrocaudal extent of the labeled and recovered axon of LCNs was 2,312 ± 113 μm (Table 2), which corresponds to two or three lumbar spinal segments in animals at this age (Fig. 3B). Mediolateral and dorsoventral spread of the axon was 636 ± 27 μm and 295 ± 41 μm, respectively. The extent of the axon of flattened and multipolar cells was similar in all dimensions, but the axons of the other five neurons (fusiform, pyramidal, and three not classified) were somewhat smaller (Table 2). The longest rostrocaudal extent encountered in this study was 6,500 μm (red neuron, cell ID: L292_E1, in Fig. 3A). It has to be noted, however, that in 51 of the 82 LCNs the axon reached one or both ends of the preparation and was therefore cut.

We used the neurons reconstructed in 3-D to compare total axon length, volume, and branch points between LCNs and ALT-PNs. For the comparison, we used a characteristic, simple lateral–collateral type (LCT) ALT-PN from Luz et al. (2010) and additionally reconstructed a mixed-collateral type (MCT) ALT-PN that was filled along with two LCNs in the same spinal cord and, to date, has the most extensively branching ipsilateral collaterals in our ALT-PN sample (blue neuron, cell ID: L292_E4, in Fig. 3A). The filling time of the three neurons was almost identical, 28–30 minutes, although the transport time of biocytin varied. The mean total axon length (i.e., sum of the lengths of all reconstructed axon segments) of LCNs was 32,717 ± 4,008 μm, which was comparable to the axon length of a complex MCT ALT-PN but much larger than that of the simple LCT ALT-PN (Fig. 3C). The mean total axon volume (calculated from the length and diameter of all reconstructed axon segments) of LCNs was 2,180 ± 378 μm^3^, a value that falls between that of a complex MCT ALT-PN and the simple LCT ALT-PN (Fig. 3D). The mean number of branch points along the LCN axon was 247 ± 71, exceeding the value of even the most complex ALT-PN axon (Fig. 3E).

**Figure 4:**
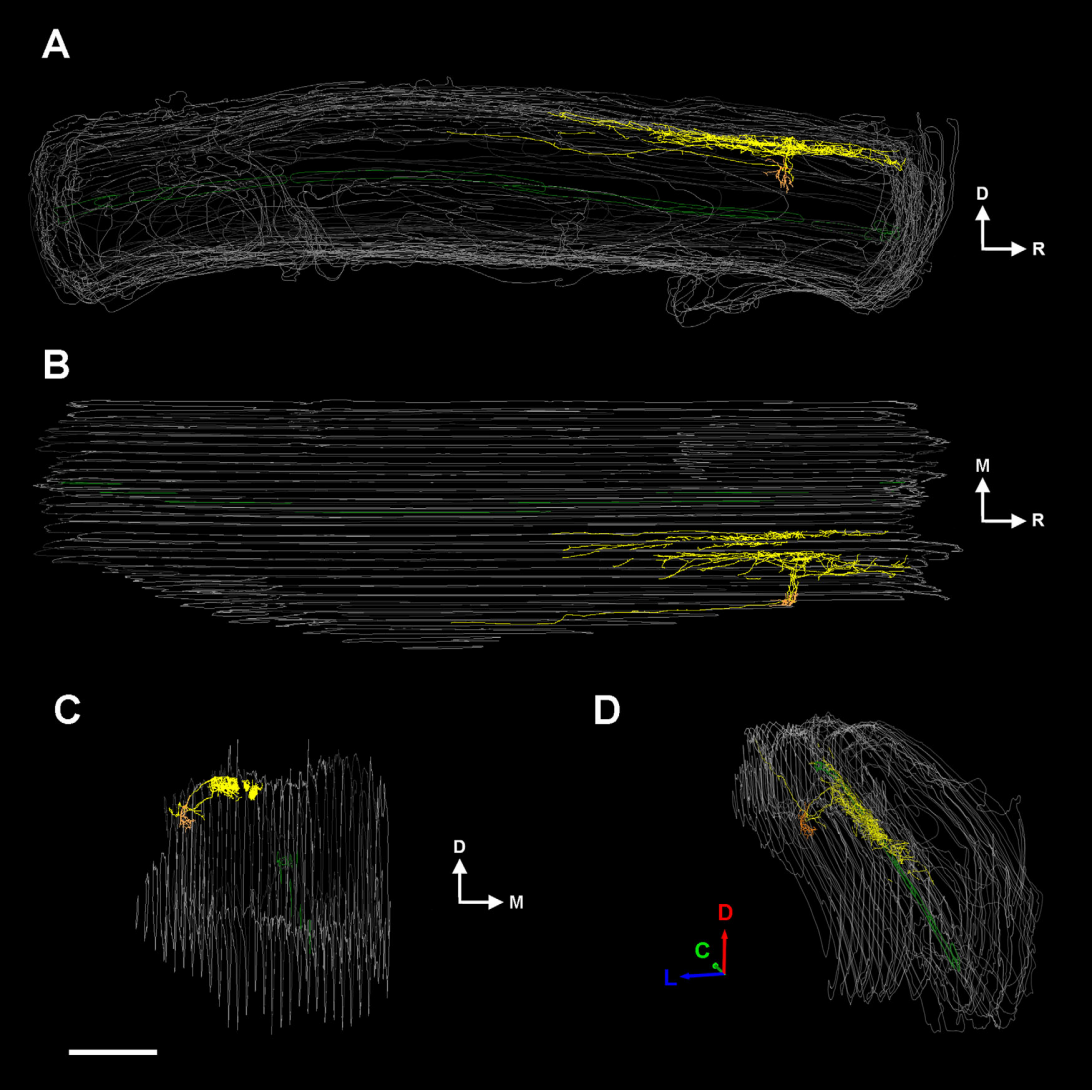
Sagittal (**A**), horizontal (**B**), transverse (**C**), and perspective (**D**) view of a 3-D reconstructed but not fully connected LCN (cell ID: Zs079_E13). The majority of the axon (yellow) in this case is located medially, considerably remote from the lateral cell body and dendrites (orange). The neuron has a solitary branch running caudally in the dorsolateral funiculus on the ipsilateral side. The medial part of the axonal tree, because of distortion of some sections, could not be connected to form a single axon. White and gray matter borders are indicated with gray, and central canal is shown in green. Scale bar = 1 mm.

**Figure 5:**
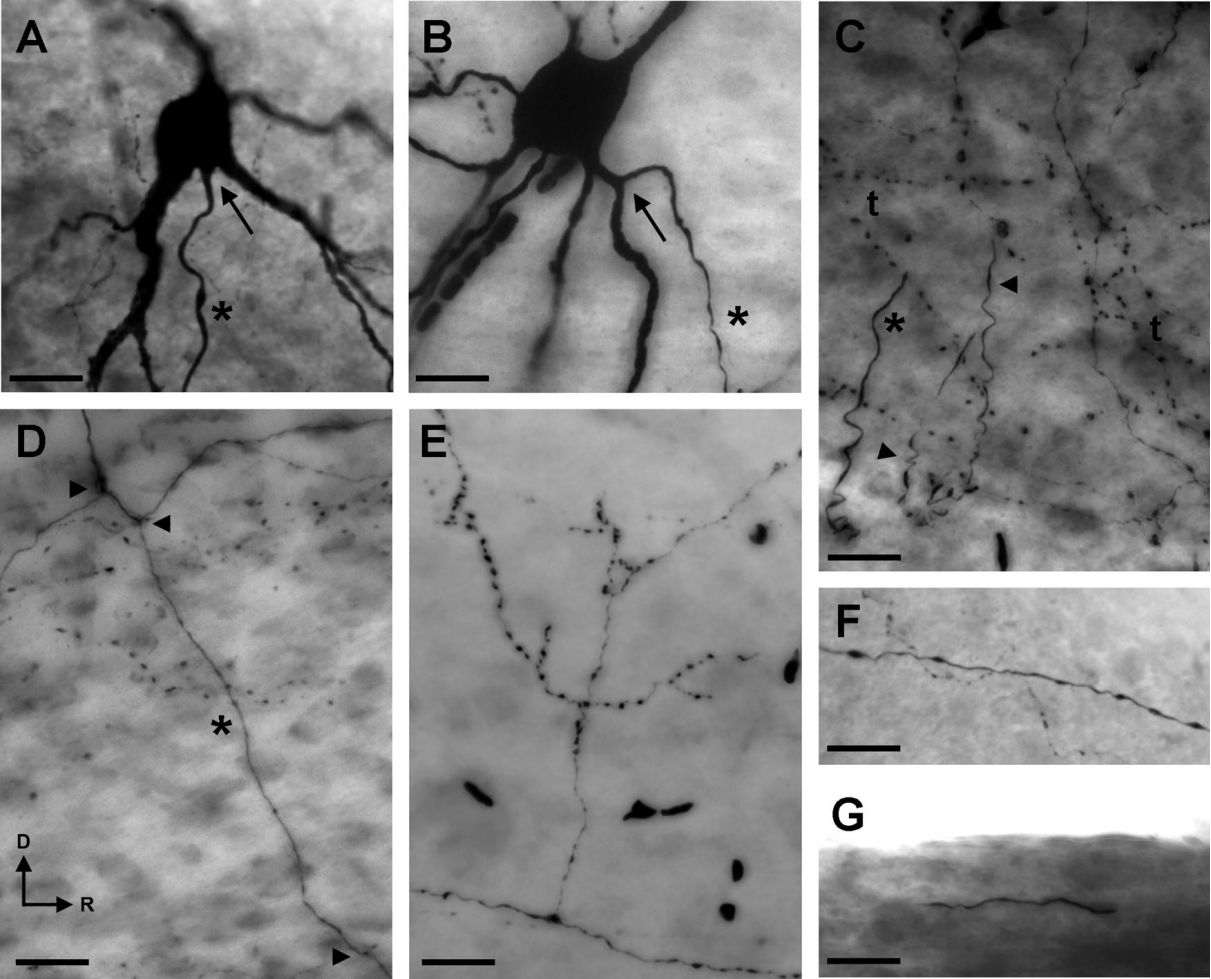
General morphological features of the axon of LCNs. Main axon (asterisk) originating from the soma (**A**) and from a primary dendrite (**B**) of two LCNs. Arrows point at the axon origin. **C**: Characteristic appearance of LCN axons in the vicinity of the cell body. The main axon (asterisk) and primary and secondary branches (arrowheads) are intermingled with fine terminal branches enriched with varicosities (t). **D**: The main axon (asterisk) of an LCN giving rise to primary branches (arrowheads) in an alternating manner. **E**: Perpendicular side branch from a solitary axon branch running in the dorsolateral funiculus. **F**: Straight, thick, solitary axon with swellings together with a more undulating, thin, varicose, also solitary branch next to the surface of the preparation, most likely in Lissauer's tract. **G**: Thick, myelinated-appearing solitary branch of an LCN, running in the dorsal funiculus. A–E,G: Extended focal images; F: single focal plane image. Scale bars = 25 μm.

Occasionally, LCNs, situated in the most lateral part of lamina I had the majority of their axon located medially, several tens of micrometers away from the soma (Fig. 4). The main axon of LCNs originated from the soma (Fig. 5A) only in 36% of the cases; the majority (46 of 82; 64%) branched from one of the primary dendrites (Fig. 5B) at a mean distance of 16.1 ± 1.6 μm from the soma (Table 3). The initial part of the axon had a myelinated appearance, similar to that of the main axon of ALT-PNs (Szucs et al., 2010), which persisted up to the first and second order, already thinner branches (Fig. 5C). In approximately half of the neurons, this initial part of the axon made a ventrodorsal and/or lateromedial loop, giving rise, often in an alternating manner (Fig. 5D), to three to seven branches of smaller diameter that traveled rostrally or caudally and branched extensively. The overlapping axons of several branch orders formed the characteristic axon of LCNs (Fig. 5C). Axon branches started to have varicosities after two or three divisions. Initial divisions of the branches frequently occurred at acute angles (30–50°), whereas distal branches, particularly the long rostrocaudal branches that run in the dorsolateral funiculus (DLF), gave rise to side branches in a perpendicular manner (Fig. 5E). The varicose part of the axon (i.e., most of the local axon in the vicinity of the soma) contained a great number of varicosities with a diameter between 0.5 and 1.5 μm (Fig. 5C). Apart from the local varicose axon network, 59 of the 82 LCNs had single or multiple, varicose or myelinated-appearing, solitary axon branches (Fig. 5F,G) in the neighboring white matter, including the Lissauer tract, dorsal funiculus (DF), DLF, and lateral funiculus (LF). Detailed description of the locations and numbers of these axon branches is given in Table 3. The percentage of LCNs with axon branches in the neighboring white matter was highest (86%) among cells with flattened somatodendritic morphology and smallest (54%) in case of the five LCNs that were neither flattened nor multipolar. Axon branches in the white matter run both caudally and rostrally, and we could establish neither a preferred direction in our sample nor any correlation with the location of the branches or any other descriptor of the neurons (e.g., somatodendritic type). None of the 82 LCNs in this study had detectable axon branches crossing the midline and entering the contralateral white matter.

**TABLE 3:** Morphological Description of the Axon of LCNs^1^

		Axon origin		Location of axon branches				
			
SD type	n	Dendrite	Distance (μm)	Only local within GM	Neighboring WM (thin with varicosities)	Lissauer/DF (thick, no varicosities)	DLF/LF (thick, no varicosities)	Ipsi- or contra-ALT
Flattened	29	17 (59%)	22 ± 3 (3–60)	4 (14%)	12 (41%)	9 (31%)	4 (14%)	0 (0%)
Multipolar	38	26 (68%)	12 ± 1 (4–26)	12 (31%)	14 (37%)	9 (24%)	3 (8%)	0 (0%)
Other + n.c.	5	3 (60%)	16 ± 5 (8–28)	7 (46%)	6 (40%)	1 (7%)	1 (7%)	0 (0%)
No soma recovered	10	n.a.	n.a.					
All LCNs^2^	82	46 (64%)	16 ± 2 (3–60)	23 (28%)	32 (39%)	19 (23%)	8 (10%)	0 (0%)

1 Data are presented as mean ± SEM (range or percentage). SD type, somatodendritic type determined using criteria of Lima and Coimbra (1986); n.c., not classified; n.a., not applicable for neurons when the soma was not recovered; distance, distance from soma to the axon origin point along the dendrite; GM, gray matter; WM, white matter; DF, dorsal funiculus; DLF, dorsolateral funiculus; LF, lateral funiculus; ipsi-, ipsilateral; contra-, contralateral; ALT, anterolateral tract.

2 Neurons for which the soma was not recovered (n = 10) were excluded from the analysis of the dendritic origin of the axon.

### Axon branching pattern

**Figure 6:**
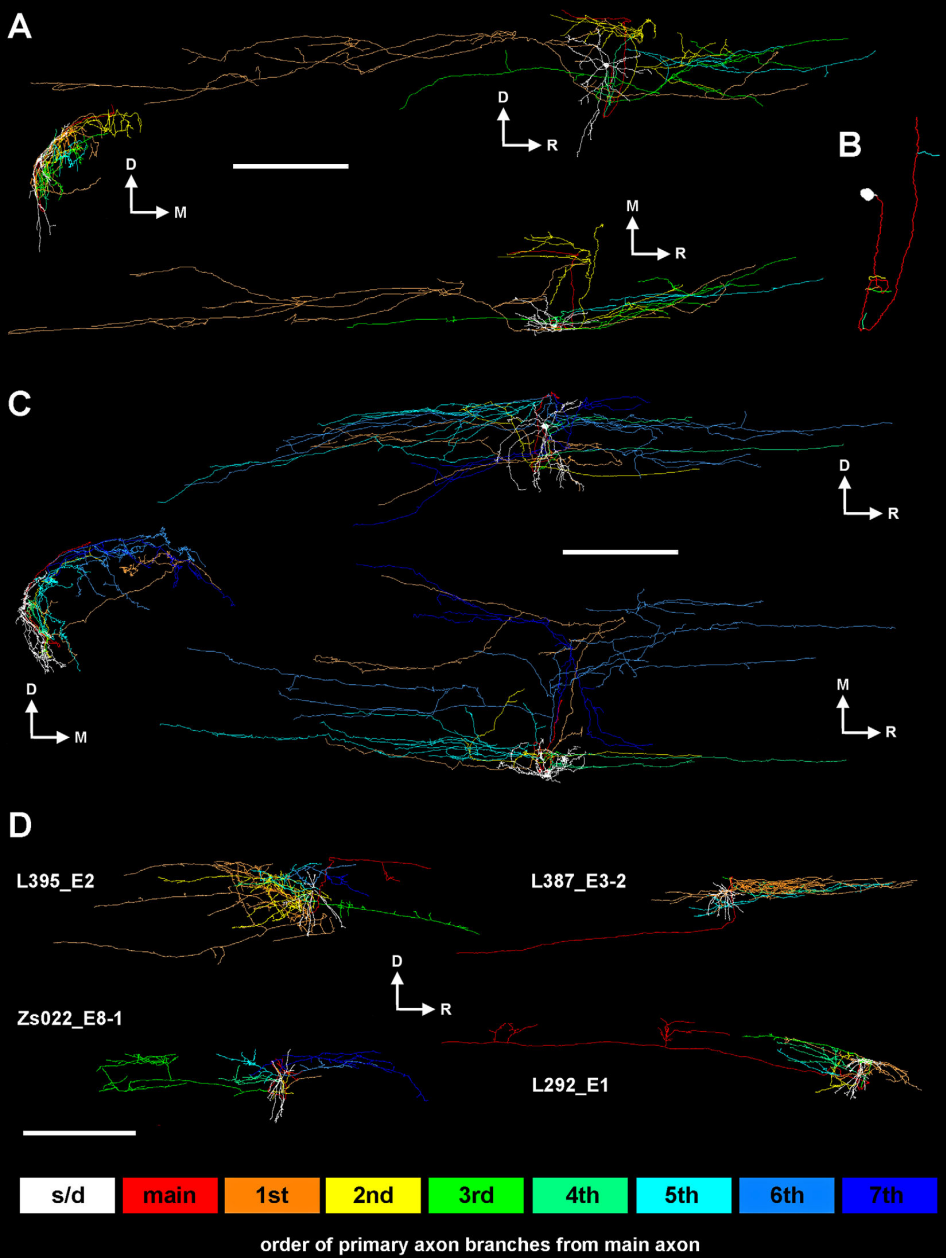
Organization of the primary axon branches of LCNs. **A**: Sagittal (top), transverse (middle left), and horizontal (bottom) view of the 3-D reconstruction of an LCN with five primary branches (cell ID: L420_E2) where the first and second branches (orange and yellow) dominate the tree, showing partial overlap with the rest of the branches. The end of the main axon turns caudally and finishes abruptly close to the surface, most likely damaged when pia mater was removed during preparation. **B**: Main axon and the origination points of each primary branch. Note the dorsoventral loop of the main axon. **C**: Another LCN (cell ID: L292_E5) with seven primary branches, from which the last two (light blue and dark blue) cover the largest area. Similarly to the previous cell, the main axon forms a loop before it finishes abruptly close to the surface with a visible swelling at the end, probably caused by truncation. The first primary branch (orange) of this axon traverses the dorsal horn medially and target similar regions than the last branch (dark blue). **D**: Sagittal view of 3-D reconstructions of four other LCNs. Cell IDs are indicated at left in the corresponding reconstruction. Colored boxes at bottom indicate the color codes of the sequence of primary axon branches. S/d, soma and dendrites; main, main axon; 1st–7th, order of primary branches from the main axon. Scale bars = 500 μm in A,C; 1 mm in D.

To understand how the intermingled axons of LCNs are organized, we color coded primary branches from the main axon of the 3-D reconstructed LCNs. In the sample of LCNs reconstructed in 3-D (n = 7), the maximum number of primary branches from the main axon was seven. We found overlaps between the target areas of the individual primary branches in all cases, despite the fact that they often started off in opposite directions in an alternating manner from the main axon (Fig. 6A,B). The overlap between target areas ranged from very slight (e.g., cell ID: Zs022_E8-1 in Fig. 6D) to substantial (Fig. 6C and also cell ID: L395_E2 in Fig. 6D). Some LCN axons had only three or four primary branches, and the proximal ones (first or second) dominated the tree (Fig. 6A). In other LCNs, the major axon gave rise to up to seven primary branches, where distal ones occupied more space (Fig. 6C). In some cases, proximal and distal primary branches targeted similar dorsal horn areas through separate routes and added further redundancy to the tree architecture (Fig. 6C).

### Axon varicosity distribution

**Figure 7:**
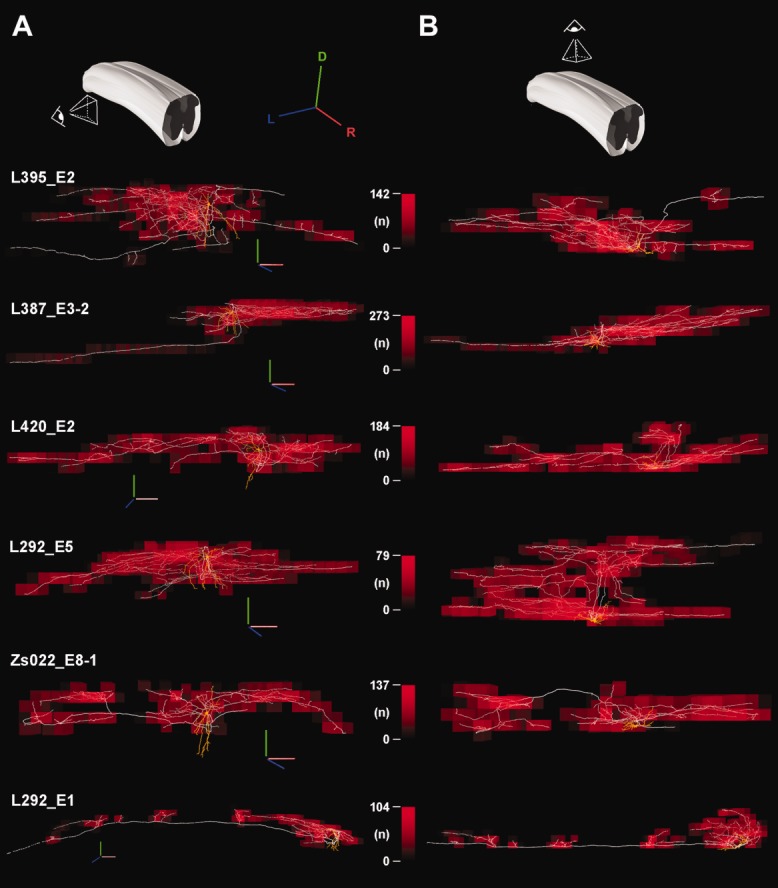
Varicosity distribution along the axon of LCNs. Spatially dependent visual representation of axon varicosity distribution from sagittal (**A**) and horizontal (**B**) views, as indicated on the schematic drawings above each column. Axon varicosities along the 3-D reconstructed LCN axons were counted in predetermined space units (voxels). The maximum varicosity number in the predefined voxels (100 μm × 100 μm × 100 μm) is indicated as the maximum value (red) on the scale bar next to the particular neuron. The actual number of varicosities in a voxel is used as a color and opacity value for the cube representing that voxel. Cell IDs are indicated at left. 3-D scale bars (D, dorsal, green; L, lateral, blue; R, rostral, red) = 250 μm.

**Figure 8:**
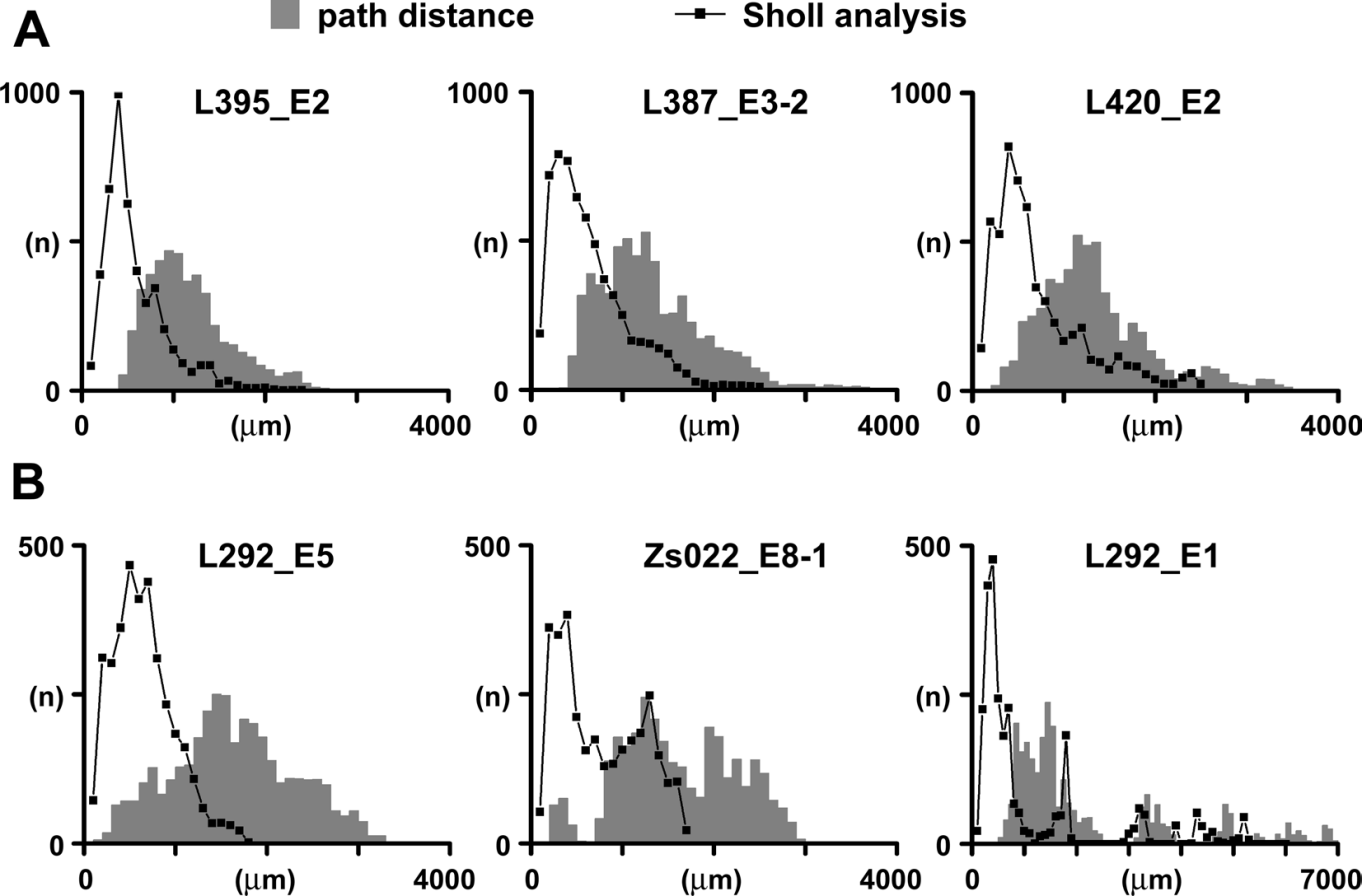
Physical and path distance of axon varicosities in 3-D reconstructed LCN axons. Sholl analysis was performed using 100-μm shells. Bin size in path distance histograms is 100 μm. **A**: Three cells showing a major accumulation of varicosities in the vicinity of, but not centered on, the soma, with one peak both in the Sholl analysis and in the path distance histogram. **B**: Other LCNs, with lower overall number of varicosities, showing additional local accumulations, evidenced by multiple peaks in the Sholl analysis, and at the same time wider distribution of the path distance histogram. Note the different scaling of the Y axis between A and B and also the difference on the X axis in cell ID: L292_E1 in B.

The axonal tree of LCNs possessed a large number (up to 6,122, cell ID: L387_E3-2) of en passant varicosities and terminal boutons, typical for unmyelinated axons. The absolute number of detected varicosities in case of individual cells largely depends of the extent of labeling and the intactness of the axon, so it is likely to show substantial heterogeneity. Varicosity density and distribution along the axon, however, may show characteristic patterns even in cases of incomplete cell labeling. Thus, we performed 3-D Sholl analysis and path distance measurements, combined with mapping of varicosity distribution along the axon in a spatially dependent manner. For the latter purpose, a custom-made program was created that counted the varicosities in a predetermined space unit (voxel) and represented it visually on top of the 3-D representation of the axon. The maximum varicosity number in the predefined voxels ranged from 79 to 273 among the 3-D reconstructed LCNs, and the distribution along the axon was clearly heterogeneous (Fig. 7). As a general feature, all six neurons presented the highest number (and density) of varicosities in the vicinity (within the first 500 μm) of, but not centered on, the soma (Figs. 7, 8). The number and density of varicosities gradually decreased farther from the soma (e.g., cell IDs: L395_E2 and L387_E3-2, Figs. 7, 8A). Path distance to the individual varicosities in these cells also showed a definite peak around 1,000 μm. However, for cells that had fewer varicosities within the first 500 μm, we observed further local accumulation of varicosities that were sometimes located several hundreds of micrometers away from the soma along the rostrocaudal (e.g., cell IDs: Zs022_E8-1 and L292_E1, Figs. 7A, 8B) or mediolateral (e.g., L292_E5 in Fig. 7B) axis. In these cells, path distance histograms showed a more widespread distribution with multiple peaks (Fig. 8B).

One of the cells presented several such accumulations (cell ID: L292_E1, Figs. 7, 8B) on side branches originating from a major projecting branch that ran caudally for several segments, gradually becoming thinner. Interestingly, accumulations were spaced in a way that their position corresponded to different lumbar segments, and, while avoiding the region with dendrites of an MCT ALT-PN (cell ID: L292_E4, blue cell in Fig. 3), they overlapped with dendrites of another LCN (cell ID: L292_E5, green cell in Fig. 3). Long solitary branches that projected out of the main part of the LCN axon usually had a constant diameter and were devoid of varicosities (consistent with the appearance of myelinated axons), except for occasional side bouquets (e.g., cell ID: L395_E2, Fig. 7).

### Fine structure of LCN axons

**Figure 9:**
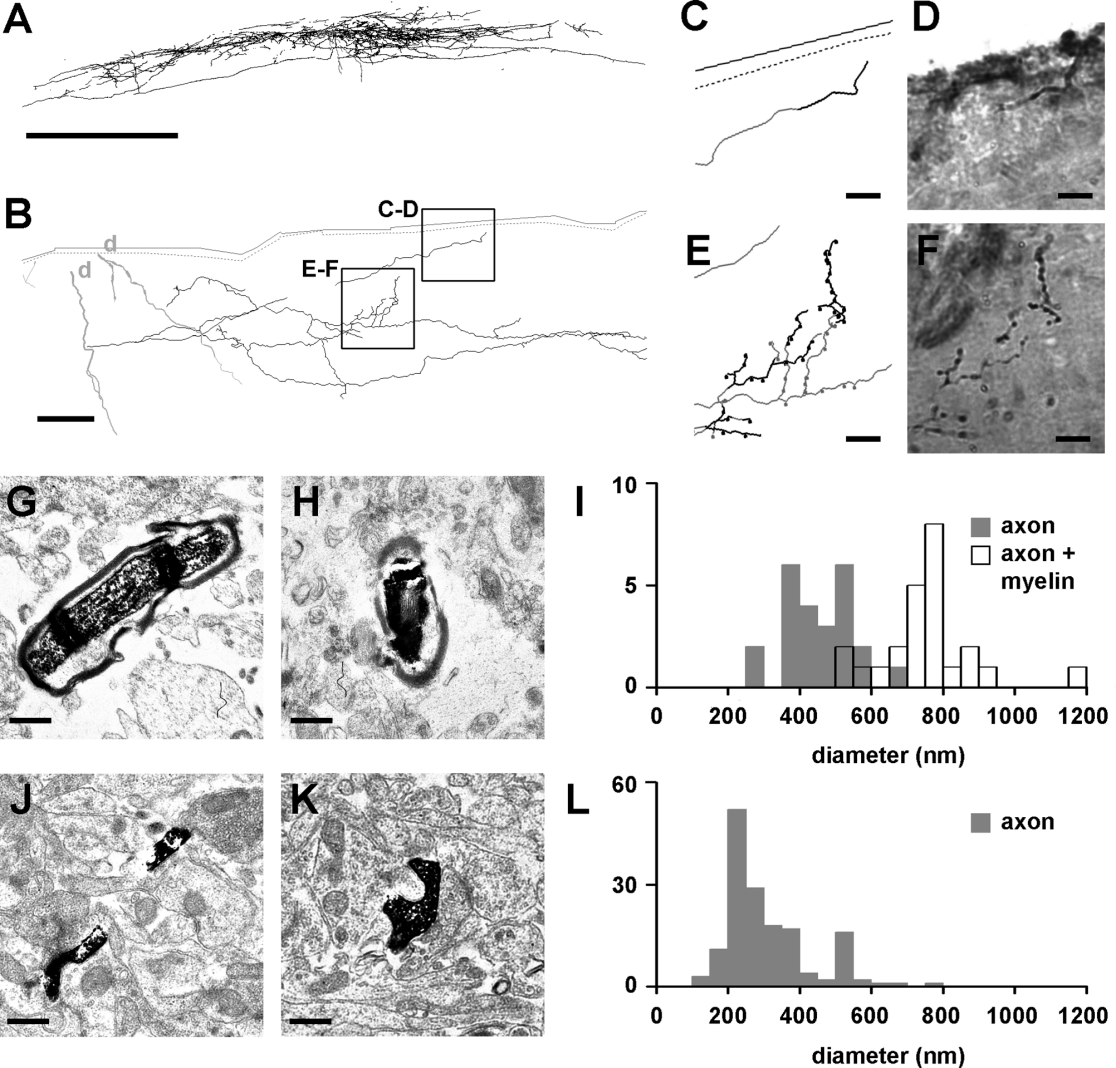
Fine-structural difference between myelinated and unmyelinated parts of an LCN axon. **A**: 2-D reconstruction of a representative LCN axon (cell ID: L279_E2). **B**: 2-D reconstruction of a single section of the same axon. Light gray processes are dendrites (d); the axon is in black. Continuous and dotted gray lines indicate the border of the section and border between the gray and white matter, respectively. Boxes indicate regions of interest with a single myelinated-appearing axon branch (C,D) and several varicose branches (E,F). **C–F** show the reconstruction of the corresponding region and a photomicrograph of the same region from the surface of the block used for preparing the electron microscopic sections. Black parts of the reconstructions in C,E show axon pieces still present on the block surface, whereas gray indicates parts that were already cut. **G,H**: Electron microscopic images of axon profiles from region C,D with several concentric layers of myelin. **I**: Diameter histogram of axon profiles (n = 24) from the same region measured with (open bars) and without myelin (shaded bars). **J**: Electron microscopic image of an intervaricosity segment and a varicosity (**K**) from region E,F. **L**: Diameter histogram of axon profiles (n = 157) from the same region. Bins = 50 nm in I,L. Scale bars = 1 mm in A; 50 μm in B; 10 μm in C–F; 500 nm in G,H,J,K.

Because the axon of LCNs could clearly be divided into varicosity-bearing thin parts and thicker myelinated-appearing pieces, we wanted to confirm the absence and presence of myelin in these regions. We selected an LCN with a characteristic axon (Fig. 9A) and chose a section where myelinated-appearing thick pieces were present along with thin parts, densely packed with varicosities (Fig. 9B). The section was processed for electron microscopy analysis and two regions of interest (Fig. 9C,D, thick myelinated-appearing piece; Fig. 9E,F, thin varicose branches) were re-embedded and resectioned. We found that axonal profiles from the region shown in Figure 9C,D, without exception, presented several concentric layers of myelin around them (Fig. 9G,H). At the same time, intervaricosity segments (Fig. 9J) and varicosities (Fig. 9K) of axons from the region shown in Figure 9E and F both lacked myelin.

We also measured the diameter of the axons on the electron micrographs taken from the two regions of interest. Because the axons in the ultrathin sections were cut in a random orientation, we first established the longitudinal axis of the particular profile and then measured the widest diameter perpendicular to it. Diameter histograms of myelinated (n = 24) and unmyelinated (n = 157) axon profiles are shown in Figure 9I,L. The mean diameter of myelinated axon profiles measured with and without the myelin were 760 ± 28 nm and 453 ± 18 nm, respectively. Mean unmyelinated axon diameter was 311 ± 10 nm, with most diameters in the bin between 200 and 250 nm. The ultrastructure of the preparation did not allow identification of further fine structural details or postsynaptic targets of the LCN axon.

### Action potential propagation time in LCN axons

In an earlier study, we estimated the action potential (AP) propagation times at different points of a complex LCN axon by using an anatomically correct neuronal model, in which the whole axon was considered unmyelinated (see Fig. 8 of Luz et al., 2010; cell ID: L-395_E2). Our previous results indicated that the fine caliber and large length of LCN axons result in significant AP propagation times, up to tens of milliseconds. In the present study, however, our fine-structural analysis revealed that some parts of the LCN axon are myelinated. Thus, we wanted to test the extent to which the myelinated regions can reduce AP propagation time in the axonal tree. Furthermore, we wanted to calculate the theoretical AP propagation time for every point of the axonal tree to establish a temporal map of AP propagation in LCN axons. We developed a program in the Python programming language to represent visually (in form of a color code) the propagation times (from 0 msec [blue] to the maximum value [red]) for all points of the 3-D reconstructed LCN axons. The diameter threshold above which we considered the axon segment as myelinated has been set at 0.35 μm, based on the histograms and means of myelinated and unmyelinated axon diameters (Fig. 9I,L). Conduction velocity in the unmyelinated part was set to 0.38 m/second for a uniform 1-μm-thick axon in agreement with measurements made at 22–24°C for unmyelinated C-fibers in isolated dorsal roots (Pinto et al., 2008). For myelinated axons, we used a scaling factor giving a value of 10 m/second for a uniform 1-μm-thick axon, so that the resulting conduction velocities fell in the range of those for the nociceptor-driven lamina I projection neuron axons of the small myelinated type (3.8–16.5 m/second; Cervero et al., 1979).

**Figure 10:**
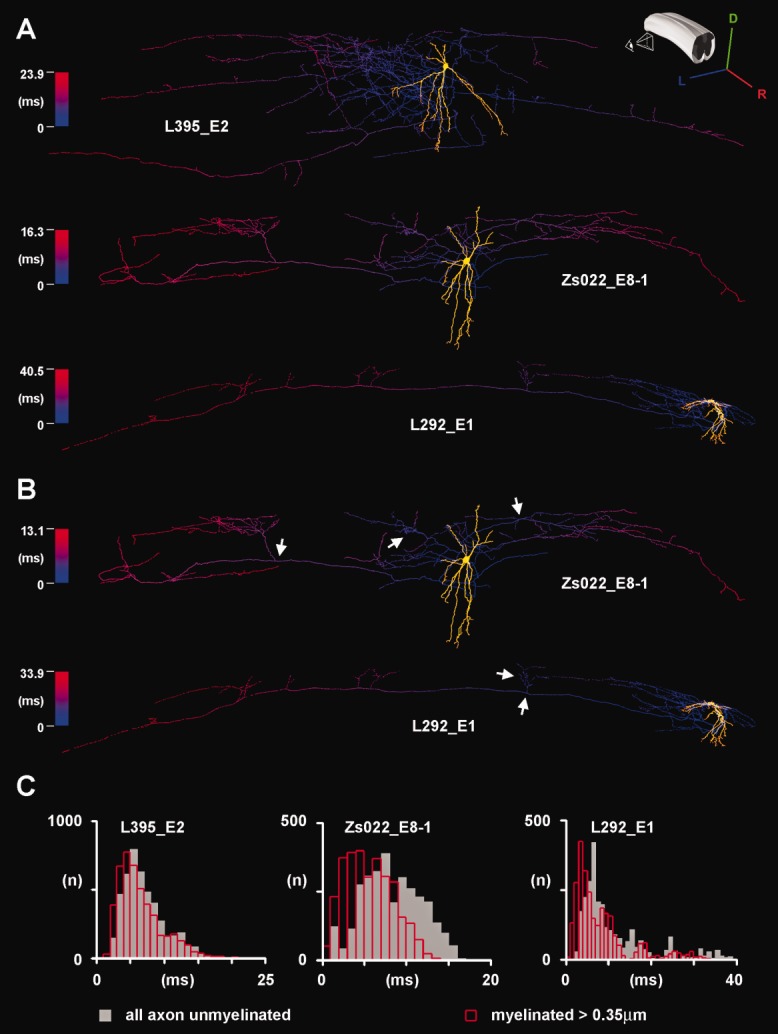
Action potential (AP) propagation time maps of LCN axons. **A**: Sagittal view (see schematic at upper right) of a cell (ID: L395_E2) with a more compact tree and two cells (IDs: Zs022_E8-1 and L292_E1) with distant terminal branching areas. The whole axon is considered unmyelinated. **B**: AP propagation time maps of two cells (IDs: Zs022_E8-1 and L292_E1) replotted assuming that axon pieces with a diameter above 0.35 μm are myelinated. The maximum propagation time value in the map is indicated by red, whereas 0 msec is blue on the scale bar next to the particular neuron. Arrows point to regions where the propagation time map shows visible differences. **C**: Propagation time histogram of the axon varicosities of the three LCNs shown in A. Bins = 1 msec. Shaded bars, total axon unmyelinated; open bars, axon pieces with a diameter above 0.35 μm are myelinated.

To create the AP propagation time maps, we chose two LCNs (cell IDs: Zs022_E8-1 and L292_E1) with remotely situated terminal branching areas and also included, as a form of validation of the program, the LCN used in our earlier work (cell ID: L395_E2; Luz et al., 2010). First we calculated conduction velocities for the axons considering them all as unmyelinated (Fig. 10A). The propagation times for cell L395_E2 were in the same range as those calculated using our former method (Luz et al., 2010). In the case of the LCN with the longest solitary branch (cell ID: L292_E1), the maximum propagation time in the remote regions was above 40 msec. In all three modeled LCNs, the propagation time gradually increased in a concentric manner from the soma toward peripheral parts of the axon.

When propagation times were recalculated considering axon segments with a diameter above 0.35 μm as myelinated, the maximum propagation times in each neuron dropped (Fig. 10B). For the LCN with a compact tree, lacking remote regions attached via thick, long, solitary branches (cell ID: L395_E2), the change in the maximum propagation time was negligible (about 2%, 23.9 msec changed to 23.5 msec). The propagation time distribution of the axon varicosities was only slightly shifted to the left, with no apparent change in the shape (Fig. 10C).

For the other two neurons (cell IDs: Zs022_E8-1 and L292_E1), however, maximum propagation time dropped to about 80% of the original value (to 80.4% in case of Zs022_E8-1 and 83.7% in L292_E1). Changes in the propagation time map were most apparent in case of Zs022_E8-1 (see arrows in Fig. 10B). Propagation time distribution histograms of axon varicosities of the latter two LCNs were notably shifted to the left, and their temporal dispersion decreased (Fig. 10C).

### Neurotransmitter content of LCN axon terminals

**Figure 11:**
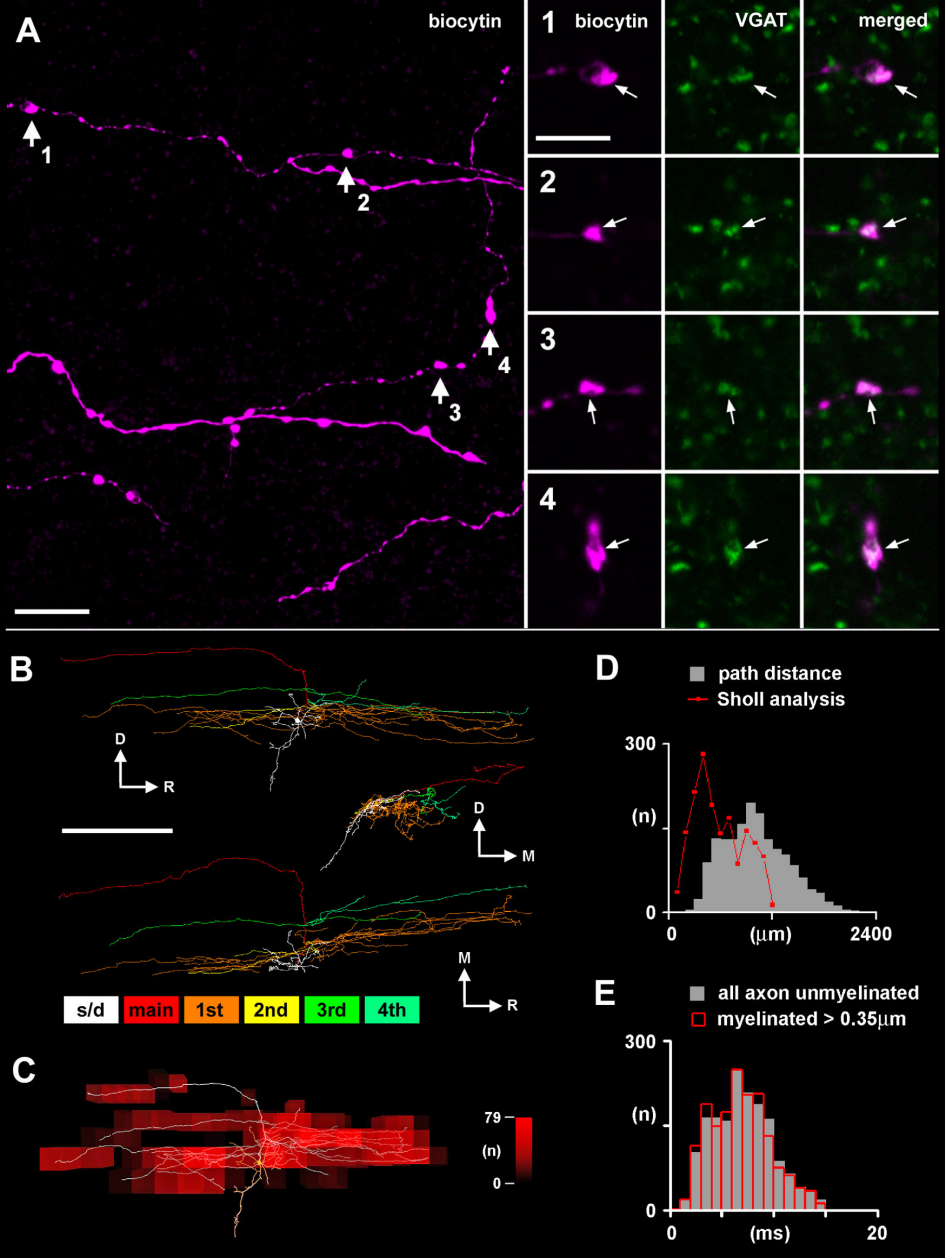
Example of an inhibitory LCN. **A**: Single section of a biocytin-filled axon (magenta) of an LCN (cell ID: L571_E20-3). Arrows with numbers indicate the VGAT-immunoreactive axon varicosities (green) shown in detail in the **insets** at right. Arrows in insets point to the actual varicosity. The main image is projected from 36 optical sections at 0.5-μm Z spacing, whereas images in the insets are single optical sections. **B**: Primary branch organization of the same LCN axon shows relatively little overlap among the branches. **C,D**: The number of axon varicosities in this axon is moderate, and varicosity distribution shows more than one, relatively dispersed accumulation. **E**: Temporal dispersion of action potential propagation times in the same LCN axon did not show a noticeable difference when using the diameter threshold for myelinated axons, indicating the lack of long myelinated parts in the axon. Bins = 100 μm in D; 1 msec in E. Scale bars = 10 μm in A; 5 μm in insets; 500 μm in B.

A question remaining after the description of the complex axon of LCNs was whether these neurons are excitatory or inhibitory. To answer this question, with 18 LCNs we tested immunoreactivity of the axon varicosities for antibodies raised against VGAT and VGLUT-2 (Yasaka et al., 2010). In 16 LCNs, axon varicosities showed intensive immunolabeling for VGAT (Fig. 11A) and no sign of the presence of VGLUT-2 (not shown). In one LCN neither VGAT nor VGLUT-2 was detectable, and in another case the reaction was weak and immunopositivity could not be determined. To confirm that both transporters are indeed detectable in our preparation, we tested their presence in dorsal, lateral, and ventral collaterals of excitatory ALT-PNs (n = 4). Axon varicosities of the collaterals, as expected, proved to be VLGUT-2 immunopositive (images not shown). The mean soma area of the inhibitory LCNs, 246 ± 8 μm^2^ (n = 6 somata recovered) was in the range of smaller cells in the LCN group (see Fig. 1G). Inhibitory LCNs belonged to the multipolar (n = 5) and flattened (n = 3) somatodendritic types (for cells in which dendrites were recovered sufficiently for classification). The axon of the reconstructed inhibitory LCN (along with the rest of the analyzed inhibitory LCN axons) was similar to those of the rest of the LCN sample (Fig. 11B–E). This particular cell had an axon that showed accumulations of varicosities both on the caudal and on the rostral parts of the tree (Fig. 11C).

## DISCUSSION

We used an intact in vitro spinal cord preparation to demonstrate the axon diversity of LCNs in lamina I. We provide morphological evidence for their possible involvement in segmental interlaminar and propriospinal sensory information processing by showing that LCNs frequently possess a number of short and putative long propriospinal branches. To the best of our knowledge, for the first time, we provided extensive 3-D reconstructions of several lamina I neurons that allow novel topological analyses. Besides the complexity of LCN axons, these analyses also revealed heterogeneity in the overlap between major axon branches, in the distribution of axon varicosities, and in the estimated AP propagation times for different points of the axon.

### Technical considerations

Our sample in this study is biased in some respects. First, we used young animals, because the viability of the in vitro intact spinal cord preparation in our experiments proved to be best at P14–P24. For this reason, one cannot exclude that axons of more mature LCNs may be different. This, however, seems unlikely, in that lamina I neurons with very similar local axon branching pattern have been described for adult rats (Li et al., 2000). Second, because of the decreased visibility through regions rich in myelinated fibers, the medial part of the dorsal horn surface (dorsal root entry zone) was excluded from the search for neurons. Thus, conclusions of this study are based on neurons from the lateral two-thirds of lamina I. Third, when we visualized the surface of the dorsal horn in our intact spinal cord preparation, detection of the laminar border between lamina I and substantia gelatinosa relied solely on the appearance of the uniform, densely packed layer of small lamina II neurons. For this reason, to ensure that recorded neurons were in lamina I, we selected larger neurons in the most superficial (first to appear) cell layer of the dorsal horn. In this way we unavoidably excluded small lamina I neurons. The description of axons of small lamina I and lamina II neurons will be the subject of another study. Finally, full analysis of all investigated parameters was possible only in 3-D reconstructed neurons. However, the number of such reconstructions is relatively small compared with the total of the LCN sample, resulting in an additional source of potential variance.

### Somatodendritic architecture of LCNs

Based on their somatodendritic architecture, lamina I neurons in the rat have been classified into four categories: fusiform, pyramidal, flattened, and multipolar (Lima and Coimbra, 1986). Lamina I LCNs in this study, with few exceptions, fell into the flattened and multipolar somatodendritic categories. This is in good agreement with our earlier study on local collaterals of lamina I ALT-PNs (Szucs et al., 2010) in which none of the filled large multipolar neurons had an ascending axon in the contralateral anterolateral white matter.

Although lamina I neurons have the bulk of their dendritic arbors confined within this lamina (Gobel, 1978), we also showed in this study that multipolar neurons issue prominent ventrally protruding dendrites (Lima and Coimbra, 1986) that not only enter lamina II, as reported for human lamina I neurons (Schoenen, 1982), but also reach lamina III. Therefore, it is reasonable to assume that, in addition to the common sources of input to lamina I neurons, multipolar cells integrate information from deeper laminae (e.g., from local axons and primary afferents terminating in laminae II and III) and should be treated as a separate group. Along this line, one cannot exclude the possibility that multipolar lamina I LCNs may even be activated monosynaptically by primary afferents that convey information to deeper laminae (e.g., A-beta afferents or A-delta hair follicle afferents; Todd, 2010). A detailed description of primary afferent input to lamina I LCNs that may answer this question is the subject of an ongoing study in our laboratory.

Similar to ALT-PNs (Szucs et al., 2010), a large percentage of LCN axons originated from one of the primary dendrites, although the mean distance from the soma in case of LCNs was slightly less. This, however, may be simply related to the fact that dendritic spread of LCNs was generally smaller than that of ALT-PNs reported from our previous study (Szucs et al., 2010). The high proportion of axons with dendritic origin among LCNs suggests that this axon initiation type is a common feature of large lamina I neurons and is in agreement with earlier reports (Cheunsuang and Morris, 2000; Hylden et al., 1986). Dendritic origin of the main axon has also been observed in cat preganglionic sympathetic neurons (Morgan, 2001) and in motoneurons (Duflocq et al., 2011). This anatomical variation seems to be frequent in spinal cord neurons, and determining its functional importance will require further in-depth knowledge of the ultrastructure and ion-channel composition of the axon initial segment and the dendrites giving rise to them (Duflocq et al., 2011).

### Overlaps between LCN axons and ipsilateral collaterals of lamina I ALT-PNs

LCNs reported in this study all had collaterals located in the superficial dorsal laminae (I–II, occasionally entering III and IV), but only about one-third of ALT-PNs (32.5%; lateral and mixed collateral types) have ipsilateral collaterals in the same region (Szucs et al., 2010). This suggests that the influence of LCNs in laminae I–II is greater than that of the ALT-PNs. In the DLF, the contribution to rostrocaudally oriented varicose axon collaterals from the two groups is more balanced: 39% of LCNs and 40% of ALT-PNs (lateral and mixed collateral types) have collaterals in this region. Thus, both types of lamina I neurons may relay local segmental information to neighboring segments and to neurons of the lateral spinal nucleus. Finally, information from lamina I to ventral laminae (V–VIII) is mostly relayed by collaterals of ventral- and mixed collateral-type ALT-PNs (30%), insofar as only a few LCNs (n = 2; 2.4%) had axons reaching lamina VII.

### Possible roles of the local axon of LCNs

The extensive branching of LCN axons suggest that, besides the anatomical divergence of primary afferent fibers, LCNs may provide further divergence of processed primary afferent information after integrating it with other sources of input. Furthermore, LCN axons occupy most dorsal laminae (I–II and occasionally III–IV), and this feature, theoretically, allows relaying of C-fiber information to deeper laminae. Indeed, Braz and Basbaum (2009) reported neurons in deep laminae (III–V) receiving polysynaptic input from unmyelinated primary afferents. Lamina I LCNs may be direct sources of such polysynaptic input or provide it through contacting lamina II islet and stalked neurons that were shown to project to deeper laminae (Eckert et al., 2003). At the same time, multipolar LCNs may be monosynaptically activated by primary afferents that terminate in deeper laminae. Consequently, neurons in laminae III–V may integrate direct primary afferent information with indirect processed form of the same information through lamina I LCNs.

Because of their extensive local axon, relatively few LCNs could influence the superficial dorsal horn along several segments, in a sustained tonic manner based on their firing pattern and the frequent occurrence of rhythmic intrinsic firing. The large number of varicosities and the highly branched, extensive axon of LCNs also imply that these neurons may be involved in volume transmission. In the case of inhibitory LCNs, rhythmic intrinsic activity may result in continuous GABA release, leading to metabotropic GABA_B_ receptor activation in neurons of several neighboring segments.

Although the majority of the tested LCN axons proved to be VGAT positive, for several reasons, it is likely that not all LCNs recorded in this study are inhibitory. About 75% of neurons in lamina I of the rat spinal cord are neither GABA nor glycine immunoreactive, and these cells (many of which are LCNs) are thought to be glutamatergic (Polgar et al., 2003). Soma areas of inhibitory LCNs fell in the lower half of the total LCN soma area range, so LCNs with larger somata may be the excitatory ones. In addition, in a previous study, we recorded synaptically connected lamina I neuron pairs, in which the presynaptic neurons were always located in lamina I, and those connections were, without exception, excitatory (Luz et al., 2010). Furthermore, about one-third of the LCNs in this study expressed functional NK1 receptors (data not shown here), which were shown to be expressed in high percentage on lamina I projection neurons (Spike et al., 2003; Todd et al., 2000) and in excitatory interneurons (Littlewood et al., 1995), although the existence of NK1 receptor positive/GABAergic neurons has also been proposed for the spinal (Ferrini at el., 2007) and the medullary (Wang et al., 2000) dorsal horn of rats.

We have demonstrated that, although the general appearance of the complex local axon is similar in all LCNs, axon varicosity distribution and branching pattern show certain heterogeneity. This may be related to the segmental position or to the location of a particular LCN in the somatotopic map. The relatively low number of 3-D reconstructed axons that could be used for such topological analyses, however, did not allow us to establish such correlations.

### Functional consequences of the organization of LCN axons: branching, varicosity distribution, and spike propagation

The complex axons of LCNs with large numbers of varicosities and a tonic firing pattern, together with the observation that some of these neurons are rhythmically active (data not shown here), suggest that LCNs could provide tonic regulation and could distribute integrated information in a pattern governed by inherent spatial and temporal properties of the axon. For a complex axon with thousands of varicosities, forming networks in a highly somatotopic spinal dorsal horn, temporal dispersion of synaptic output is of crucial functional importance. The propagation time of an AP, initiated in the axon initial segment, is determined mostly by path length, axon diameter, myelination, and branching (Debanne et al., 2011). Path distance to a particular point of the axon is strongly dependent on branching. The alternating branching of primary collaterals from the main axon into the rostral and caudal directions, frequently observed in this study, seems to be an efficient solution for maximally filling the target space (laminae I–III) and, at the same time, equalizing path distance for the rostral and caudal portions of the axon tree. This setup, however, in a system with strong spatial boundaries, such as the spinal dorsal horn, results in an overlap between major branches. Target regions supplied by multiple overlapping branches may be activated repeatedly, so it will be important to understand conduction properties of LCN axons to evaluate their role in the spinal network.

A recent article comparing 3-D reconstructed basket and spiny cell axons used uniform conduction velocities to estimate, among other things, temporal dispersion in the axonal trees (Budd et al., 2010). The authors pointed out that this approach might underestimate temporal economy, because, for example, myelinated primary axon collaterals could reduce latency to child branches with the same amount of axon for wiring. Myelinated long-range branches of GABAergic interneurons, running in the stratum moleculare between innervation regions, have been shown recently in the hippocampus (Jinno et al., 2007). This study also demonstrated the presence of myelin around thicker initial parts of the axon of LCNs, and, indeed, when we used a diameter threshold to distinguish myelinated parts of LCN axons, temporal dispersion of AP propagation in the tree was reduced. This effect seemed to be more prominent in cells with remote varicosity fields connected by thicker, probably myelinated, axon pieces.

Although in our simulation of AP propagation in the complex axon of LCNs we considered the presence of myelin, this is still a simplified approach. Unfortunately, experimental data on other important factors (e.g., voltage-gated ion channel types and densities), with the exception of some parts of substantia gelatinosa neuron axons (Safronov, 1999; Safronov et al., 1997), are not available for different parts of the axons of spinal neurons. The diameter threshold for myelinated axons (0.35 μm) was chosen from measurements in a single neuron, but it is in agreement with the diameter reported for unmyelinated cortical axons (0.08–0.4 μm; Berbel and Innocenti, 1988; Westrum and Blackstad, 1962). It should also be noted that our simulated AP propagation times in LCN axons are probably overestimations, because the constants used for calculating nonmyelinated segment conduction velocities are based on measurements at room temperature. In a living organism, the AP propagation times must, therefore, be shorter. Our goal, however, was to prove that the adequately positioned myelinated regions of the axon significantly alter the AP propagation times in remote parts of the axon tree.

APs traveling through several sudden diameter irregularities such as axon varicosities have been suggested to suffer additional delay (Manor et al., 1991), and passing through several branch points may even result in a failure of propagation (Debanne et al., 1997). In the case of LCNs with thousands of varicosities, this could mean a significant further increase in propagation times and may even cut off some parts of the axon tree from signal invasion temporarily.

Subthreshold signal propagation within LCN axons could also lead to robust functional differences in different parts of the tree. Recent direct recordings from axonal structures in the hippocampus and neocortex suggested that subthreshold graded signals propagate down the axon over distances of up to 1 mm. At certain synapses, these analog axonal signals were shown to modulate AP-dependent transmitter release (Alle and Geiger, 2008). The same mechanism in LCN axons could modulate synaptic output at varicosities in the vicinity of the cell body as opposed to remote varicosities. For example, the SP-induced depolarization observed in almost half of the LCNs (data not shown here) could also propagate down the LCN axon and may boost transmitter release. Given the complexity of LCN axons and their accessibility resulting from their superficial location, they could be ideal targets for studies on axon conduction by using novel imaging techniques, such as high-speed fluorescent sodium imaging with high temporal and spatial resolution (Fleidervish et al., 2010; Foust et al., 2010), in vitro or in vivo.

### Long propriospinal connections of lamina I LCNs

Although LCNs clearly do not project through the lateral funiculus on the contralateral side, for several reasons it seems likely that they may also function as short and long propriospinal neurons. As we report here, a large percentage of LCNs (86% of flattened and 69% of multipolar neurons) had long, solitary axon branches, often with a myelinated appearance, in the DF, DLF, and Lissauer's tract. These branches had no preferential direction and often ran for two or three segments in the rostral or caudal direction before fading below visibility and were similar to those described from earlier anatomical studies (Cajal, 1909; Lenhossek, 1895; Szentagothai, 1964).

The presence of such collaterals is also in line with electrophysiological observations. About one-third of nociceptor-driven lamina I neurons could be antidromically activated by stimulation of Lissauer's or deeper tracts from up to three segments rostral to their origin (Cervero et al., 1979). The conduction velocity of the axons of these neurons suggested that they are small and myelinated (Cervero et al., 1979). In addition, unmyelinated propriospinal axons were also observed in the DLF at the sacral level (Chung et al., 1988). These latter fibers, as we showed earlier (Szucs et al., 2010) and also in this study (see blue neuron, cell ID: L292_E4, in Fig. 3), could also include lateral collaterals of ALT-PNs. Given all this, it is reasonable to say that a substantial proportion of axons in the DLF is indeed propriospinal fibers and forms massive intersegmental connections. These connections are formed not only by axons of lamina II–III neurons, resulting in a closed system (Szentagothai, 1964), but also by collaterals of lamina I neurons, some of which serve as major output elements of the dorsal horn circuitry.

Lenhossek (1895), Cajal (1909), and Szentagothai (1964) described lamina II–III neurons with several segment-spanning, rostrocaudally oriented axons in Lissauer's tract, and intersegmental integration was also proposed recently for dorsal horn cholinergic neurons, with similar rostrocaudal axonal organization (Mesnage et al., 2011). However, some LCNs in our study possess morphological features that would also allow them to establish not only short but also long propriospinal connections. Solitary branches in the DF, DLF, and Lissauer's tract never crossed the midline and ran rostrally until they faded or reached the end of the spinal cord block, providing an anatomical substrate for such long propriospinal projections. Indeed, previous reports suggested that about one-fourth of lamina I neurons have long, ascending propriospinal projections that extend from the lumbar to at least midthoracic spinal levels but do not reach the brain (Bice and Beal, b) Thus, the long, solitary branches observed in some of our LCNs may be long propriospinal branches, indicating that at least some LCNs may have influence on distal spinal cord regions. To test this hypothesis further, careful morphometric analysis of LCN axons will be needed combined with retrograde labeling from distal spinal cord segments. Eventually, understanding of local connections and outputs of lamina I neurons will allow designing better strategies for intercepting pain signals at the spinal cord level.

## ROLE OF AUTHORS

All authors had full access to all the data in the study and take responsibility for the integrity of the data and the accuracy of the data analysis. Study concept and design: PS, PA, AT. Acquisition of data: LLL, PS, RP, SYXT, AT, ZA. Analysis and interpretation of data: PS, PA, SYXT, AT. Drafting of the manuscript: PS. Critical revision of the manuscript for important intellectual content: PS, BVS, AT, PA. Obtained funding: BVS, AT. Study supervision: PS.
